# Supplementary material for: Milliwatt terahertz harmonic generation from topological insulator metamaterials
Source: Light Sci Appl. 2022 Nov 1;11:315. doi: 10.1038/s41377-022-01008-y (PMC9622918; doi:10.1038/s41377-022-01008-y)
Supplement: Supplementary file 1 — Supplement [file 41377_2022_1008_MOESM1_ESM.pdf]

Supplementary Information

for

Milliwatt terahertz harmonic generation from topological insulator metamaterials

Klaas-Jan Tielrooij[1,2,*], Alessandro Principi[3], David Saleta Reig[1], Alexander Block[1], Sebin Varghese[1], Steffen Schreyeck[4], Karl Brunner[4], Grzegorz Karczewski[4,5], Igor Ilyakov[6], Oleksiy Ponomaryov[6], Thales V. A. G. de Oliveira[6], Min Chen[6], Jan-Christoph Deinert[6], Carmen Gomez Carbonell[1], Sergio O. Valenzuela[1,7], Laurens W. Molenkamp[4,8], Tobias Kiessling[4], Georgy V. Astakhov[6,†], and Sergey Kovalev[6,‡]

[1] Catalan Institute of Nanoscience and Nanotechnology (ICN2), BIST and CSIC, Campus UAB, Bellaterra (Barcelona), 08193, Spain

[2] Department of Applied Physics, TU Eindhoven, Den Dolech 2, 5612 AZ Eindhoven, The Netherlands

[3] School of Physics and Astronomy, University of Manchester, M13 9PL, Manchester, UK

[4] Physikalisches Institut (EP3), Universität Würzburg, Am Hubland, 97074 Würzburg, Germany

[5] Institute of Physics, Polish Academy of Science, Al. Lotnikow 32/46, PL-02668 Warsaw, Poland

[6] Helmholtz-Zentrum Dresden-Rossendorf, Bautzner Landstr. 400, 01328 Dresden, Germany

[7] ICREA, Pg. Lluís Companys 23, 08010 Barcelona, Spain

[8] Institute for Topological Insulators, Am Hubland, D-97074 Würzburg, Germany

[*]E-mail: klaas.tielrooij@icn2.cat

[†]E-mail: g.astakhov@hzdr.de

[‡]E-mail: s.kovalev@hzdr.de

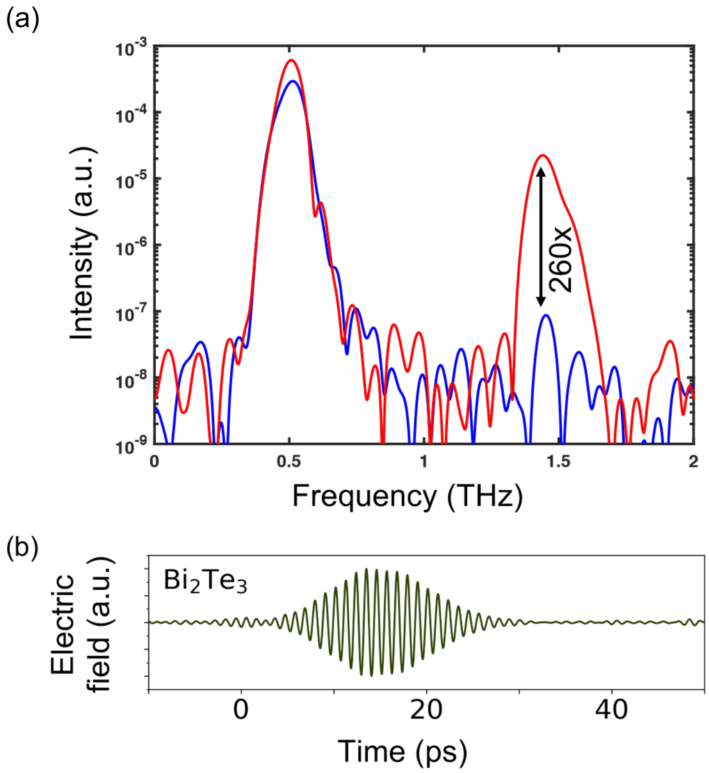

Fig. S1: Results for Bi_2Te_3. a) THz power after interacting with a 50 nm thick Bi_2Te_3 sample without grating (blue line) and with an $F = 85\%$ grating (red line), using an input field of ~300 kV cm^{-1}. The third-harmonic power enhancement is around 260, similar to the results for Bi_2Se_3 at the same input field (see Fig. 1). These measurements were performed in reflection geometry. **b)** Time-domain evolution of the harmonic signal after the 50 nm thick Bi_2Te_3 sample with grating. The dynamics show a clean envelope, similar to the results for Bi_2Se_3 (see Fig. 2). This means that – unlike the case of graphene – no saturation due to heat accumulation and heat-induced THz transparency occurs. The fundamental frequency here was 0.3 THz.
